# Religiosity and sexual abstinence among Nigerian youths: does parent religion matter?

**DOI:** 10.1186/s12889-019-6732-2

**Published:** 2019-04-18

**Authors:** Oluwaseyi Dolapo Somefun

**Affiliations:** Demography and Population Studies Programme, Schools of Public Health and Social Sciences, Faculties of Health Sciences and Humanities, University of the Witwatersrand, Johannesburg, South Africa

**Keywords:** Religion, Religiosity, Youth, Abstinence, Sexual, Nigeria

## Abstract

**Background:**

Religion plays an important role in youth behaviours, making it a significant factor in the discourse on youth sexuality in sub-Saharan Africa. Several studies have found that religion and religiosity play an important role in the sexual behaviours of young people. However, little research in Nigeria has examined the mechanisms through which religiosity influences youth sexual behaviour and if parents’ religion moderates this relationship. Guided by the social control theory, this paper contributes to the existing literature by examining the relationship between religiosity and youth sexual behaviour.

**Methods:**

Data for the study came from 2399 male and female youth aged 16–24 years in four states purposively selected from four regions in Nigeria. Abstinence was the sexual behaviour of interest. Logistic regression was used to examine this relationship.

**Results:**

Results showed that 68% of the youth had never had sex. Religiosity was a protective factor for youth sexual behaviour and this positive association was still evident even after controlling for other covariates. Youth who were highly religious (OR – 1.81, CI- 1.13-2.88) had significantly higher odds of abstaining compared to their counterparts who were not religious.

**Conclusion:**

Religiosity is a protective factor for sexual abstinence among youth in Nigeria. Policy makers can work around using religious institutions for behavioural change among youth in Nigeria.

**Electronic supplementary material:**

The online version of this article (10.1186/s12889-019-6732-2) contains supplementary material, which is available to authorized users.

## Background

There is a ubiquitous belief across Nigeria that the country is facing a decline in traditional values [1], which is leading to an increase in the number of young people engaging in risky sexual behaviours, teenage pregnancy rates, drug abuse and school drop outs. For instance, an increasing number of youth are engaging in sexual activity with multiple partners at the same time with evident gender differentials where males tend to have more sexual partners at the same time compared to females [2]. These risky behaviours have been linked with factors at the individual, family and environmental levels, which have been noted to put young people at risk of sexually transmitted infections and may affect them throughout their life course [3].

Despite the focus on risky behaviours among youth in Nigeria [2, 4], some young people are able to engage in protective behaviours and have positive outcomes even in the face of exposure to factors that have been shown to favour risk, such as social media and unsupportive family structure [5]. The presence of such youth has inspired research in resilience.

Resilience has been described as surviving difficult situations in one’s life and being able to exhibit positive outcomes after the said event [6]. Studies have linked resilience to a number of protective factors at the individual’s disposal. Protective factors could be the internal assets of the individual or external factors in the environment that buffer risk [7]. Documented examples of internal assets include having positive self-esteem, being goal oriented, and being socially responsible. External factors consist of supportive environments and family structure, which are essential for having a strong social network and usually influence the level of support one can receive in the environment [8].

As part of this growing body of resiliency research, religious participation and belief in a spiritual power have been identified as protective factors in a number of adverse environments [9]. Religion is a contemporary factor in the lives of many young adults in sub-Saharan Africa [10–12]. In addition to creating more space for youth to maintain close relationships and participate actively in a religious environment, some religions have promoted the dissemination of particular moral norms, as well as punitive sanctions, with respect to many aspects of their younger followers’ lives, including encouraging delay in timing of sexual debut until marriage. Different religious groups have different expectations and these expectations differ by religious sects. Some religious sects convey messages that promote “no sex before marriage” [13] while some sects regard condoms as a contraceptive method alone as opposed to a method that can be used for both contraception and disease prevention [14]. These different belief systems usually have varying effects on behaviour and health [15].

Religion has also been said to help young people survive difficult situations [16]. For instance, using a sample of youth in foster care, Jackson, White [17] found that majority of the youth sampled remained hopeful during hardships and were likely to pray when something bad happened to them. Another way in which young people could survive is the social capital that may be available and accessed through religious memberships. Members of religious associations may also serve as sources of support when members lose other close members [18]. This becomes a source of support for young adults in single parent households or orphans.

Although the influence of religious institutions on behavioural change among youth have been widely accepted, others have argued that these religious teachings restrict sexual discussions to sex alone and overlook socio-economic determinants of sexually transmitted infections [19]. This approach has limitations with regards to understanding the context in which behaviours occur and the factors driving them.

Quantitative studies have examined the relationship between religion and youth sexual behaviour. Using nationally representative data from nineteen countries in sub-Saharan Africa (SSA), Magadi and Uchudi [20] found that female adolescents who were Protestants were at lower risk of early sexual debut compared to their counterparts who were Catholics. Another study in Rwanda found that there were no significant differences between Catholics and Protestants practicing primary sexual abstinence among male adolescents [21]. Exploring the effect of religious affiliation on different sexual behaviours in Zambia, it was found that affiliation with some specific groups (Seventh Day Adventists, Jehovah’s Witnesses, and the New Apostolic Church) was associated with delayed sexual initiation, but not condom use at first sex, which may have implications for risk of HIV [22].

Contributing to the literature on the relationship between religion and youth sexual behaviour, Olivier and Wodon [23] reviewed studies on religion and various health outcomes. They suggested the need for better understanding of how new religious traditions impact sexual behaviours. Additionally, Shaw and El-Bassel [24] mentioned that one of the major drawbacks of studies that have examined the association between religion and sexual behaviours is that the mechanisms through which religion influences sexual behaviour has been overlooked.

Religiosity is a significant characteristic of religion, which focuses on the strength of religious beliefs and involvement. It has been defined as an individual’s belief, spirituality, and reverence towards a divinity [25].

We focus on the relationships between religiosity and abstinence because abstinence has been consistently referred to as the most effective way of avoiding STI’s and unplanned pregnancies [26]. In addition, existing literature has documented the moderating role of parent religion on youth behaviours. For instance, family religiosity had a significant association with adolescent religious attendance and sexual initiation in a study conducted in the United States [27]. Therefore, we also explore the moderating role of parent’s religion and contribute to the existing literature by examining whether the presence of parents in the household influences the association between youth religiosity and sexual abstinence.

There is a dearth of literature on such associations in SSA, which makes this study timely especially among Nigerian youth. In addition, given the notable changes that Nigeria is experiencing in the religious landscape and the large number of young people [28], research is needed that examines the association between religiosity and youth sexual behaviour. Therefore, this study aims to fill that gap by exploring the role of religiosity on sexual abstinence among young persons.

## Theoretical framework

### The social control perspective

This study uses the social control perspective to explain the mechanisms through which religiosity influences youth sexual behaviour. This theory was developed in the 1960s to explain delinquency [29] and has since been applied in the study of sexual behaviours [30, 31]. According to Hirschi and Stark [29], connections within societal institutions, such as church and family, restrain individuals from engaging in non-standard behaviours by encouraging oversight of youth and providing models for healthy behaviours. Social interactions, particularly those with family, help children to internalize shared moral norms for behaviours [32]. These interactions operate at different levels of the environment (individual, family and community). Nigeria has witnessed an unmatched increase in religious dogma in the past 30 years compared to other countries in sub-Saharan Africa [33]. Although major religious groups are limited to two, Christianity and Islam, there has been an increase in the number of denominations particularly among the Christians [34]. We therefore anticipate that interactions with religious instructions and leaders may influence youth religiosity, which may eventually influence youth sexual behaviour. We also anticipate that these interactions would be strengthened in the presence of parents in the household and with parental religious affiliation.

### Hypotheses

Our review of the literature and theoretical framework, which posits that religion must be positioned within an ecological framework [30], suggests several hypotheses. We hypothesize that:

1. higher levels of youth religiosity will be associated with sexual abstinence due to biological factors such as gender at the individual level,
2. the association between religiosity and abstinence will operate, in part, through parental religion and presence of parents in the household, which are family level factors,
3. the association between religiosity and abstinence will also be mediated by family and community social capital;
4. social capital could operate outside the family and at the community and has been measured by presence of role models in this study.

## Methods

### Study area

Nigeria is a diverse society with a population of approximately 180 million people and several religions and tribes. Nigeria is Africa’s most populous country and religion is an important phenomenon in the country that affects every segment of the Nigerian society. There are two major religions in Nigeria, Christianity and Islam, and their practice varies regionally. Cultural and geographical differences between these religious groups influence the prescribed sexual behaviours of their adherents. Protestantism and local syncretic Christianity are evident in the Yoruba areas of South West Nigeria, while Catholicism dominates Igbo and closely related areas in the Eastern part. A large number of people in the Northern part of Nigeria practice Islam and majority of people in the middle belt are Christians. Currently, there is a new wave of Pentecostalism which has attracted the young people aged 15–24 [35].

For ease of administration, Nigeria is divided into 36 states and Abuja, the federal capital territory. These states are then grouped under 6 geopolitical zones. The study took place in four states, one from four of the six geopolitical zones. The rationale for selection was purposive. These zones were selected by examining the prevalence of protective sexual behaviours among youth in Nigeria using the 2013 Nigeria Demographic and Health Surveys (NDHS, 2013). In this study, protective sexual behaviours are defined as behaviours that protect young people from risk. These behaviours include abstinence, condom use at last sex, HIV testing and single sexual partnerships.

### Study population

This study sampled unmarried male and female youth aged 15–24.

### Study design

This was a cross-sectional study that took place from February 2018 through May 2018.

### Sample size

Using the sample size for single proportions,

$$ \mathrm{n}=\frac{{\left({\mathrm{Z}}_{\upalpha}\right)}^2\left[\mathrm{p}\left(1-\mathrm{p}\right)\right]}{{\left(\mathrm{d}\right)}^2} $$

Where n is the minimum sample size

Z_α_ = Standard normal deviate corresponding to a 2 sided level of significance of 5% = 1.96

p_1_ = prevalence of positive sexual behaviour =45.7% (NDHS, 2013)

$$ \mathrm{q}=1-p=1-0.457=0.543 $$

$$ \mathrm{d}=\mathrm{desired}\ \mathrm{precision}=5\%=0.05 $$

Adding a Design Effect of 1. 2 based on cluster sampling technique = 476 × 1.2 = 571.2

Therefore, a minimum sample size of 571 youths was calculated. However, due to the population of Nigeria and the percentage of youth aged 15–24 in the country, this study’s sample size was calculated for each of the purposefully selected states using their varying prevalence rates of protective sexual behaviours. This approach has also been used by ICF international for the Nigerian Demographic and Health Surveys. The sample size for each state is presented in Appendix.

### Sampling design

The study adopted a 3-stage cluster random sampling technique. A total of 2339 young people took part in the study. Participants were drawn from four states in Nigeria. These states were selected based on their regional variability and prevalence of positive sexual behaviours in the Nigerian Demographic and Health Surveys (NDHS). The study took place in Edo, Enugu, Osun and Kano states purposely selected. Based on the 3-stage cluster sampling technique, youth were recruited thus:

### Stage 1

Each state has three senatorial districts with an average of 11 local government areas per district. The list of local government areas (LGA’s) as obtained from the Nigeria Bureau of Statistics (NBS) and was stratified into urban, sub-urban and rural. Using a simple random sampling technique, two LGAs were selected by balloting from each urban, sub-urban and rural LGAs making a total of 6 local government districts per senatorial district.

### Stage 2

In each LGA (urban, sub-urban and rural), two wards were selected by balloting from the list of wards obtained from the LGA commission.

### Stage 3

Wards were regarded as clusters. In each ward, locations where youth congregate were identified and included markets, lesson centres, garages and schools. Young people (male or female) who were within the age group 15–24 were approached and invited to participate in the study.

### Procedure

The quantitative data were collected electronically through Open Data Kit (ODK collect). The ODK collect application was installed on a set of Android™ phones. The structured questionnaire was scripted and uploaded on a ODK sever. A pre-test of the study instrument was conducted with 60 youth recruited from the Lagos state in Nigeria to correct items that were found to be confusing. Field workers were trained on how to use the devices to collect data, while a data auditor was employed to monitor routine data upload. The interviewers read the instructions to the participants and the participants were given the Android™ phones to answer the questionnaires themselves (Additional file 1). English was used to interact with the respondents as most of them had a basic understanding of English. The field workers were proficient in English and the local languages in each of the states. They were trained to help clarify issues for respondents who presented any difficulties. Participants were allowed to complete the questionnaires at their own pace. Participants were encouraged to respond to all items, and interviewers provided individual assistance to participants who required additional help. There was no monetary compensation given to the participants for participating. The survey questionnaire included modules on education, employment, socio-demographic background, religious affiliation and practices, sexual history and behaviour, HIV/AIDS awareness, parent-child relationship, social capital and neighbourhood measures. The choice of ODK instead of paper questionnaires is to reduce errors that are associated with paper questionnaires and minimise under-reporting or over-reporting of various sexual behaviours by respondents [36].

### Ethical considerations

Study procedures were approved by the Human Research Ethics Committee (Medical) of University of the Witwatersrand (H17/11/54) and the National Health Research Ethics Committee of Nigeria (NHREC/01/01/2007–16/10/2018). Each participant signed an informed consent form. Respondents were guaranteed utmost confidentiality, privacy and anonymity. Based on the age of some of the participants, permission from parents and guardians was sought and obtained to interview the participants less than 18 years. The voluntary nature of participation was stressed to both participants and parents.

### Measures

#### Dependent variable

The outcome variable is sexual abstinence. A question “have you ever experienced penetrative sex” was asked in the questionnaire with response options as 1 “Yes” and 0 “No”. This outcome was chosen as a measure of sexual behaviour as it remains the most effective for preventing both pregnancy and sexually transmitted infections, including HIV/AIDS, among youth.

#### Key independent variable

This study considered the two dominant religious affiliations in Nigeria because the 2013 Demographic and Health Survey (DHS) indicates that most Nigerians are involved in the two popular religious groups (Christianity = 51% and Islam = 41%) while less than 2% fall into no religious or traditional categories (National Population Commission & ICF Macro, 2014). Measuring concepts that have no clear definitions remains a major problem faced by a number of social scientists [37]. Religion or religiosity is one such unclear concept because a person who believes in God may not attend places of worship regularly. Some other people may attend worship centres and not believe in God.

Religiosity is a multifaceted concept comprised of actions, participation and socio-cultural norms [38]. This is also different from a persons’ religious affiliation. For the purpose of this study, which draws from others [39–41], religiosity was measured by belief, external practice, and salience. Religious belief was measured by a question that asked youth whether they believed in God or not. Responses included “yes”, “no” and “don’t know”. External practice was measured by frequency of worship. A question “how often do you attend religious service?” was asked with response options of “everyday”, “at least once a week”, “at least once a month”, “at least once a year”, “less than once a year” and “never”. Salience was measured by the importance of faith in one’s life with responses of “very important”, “important” and “not important”. We generated separate frequency scales for Muslims and Christians. Different cut-off scores are important because of the different expectations of service attendance between the two different religious. Muslims who attended religious service “everyday” were coded as highly frequent as their religion expects them to pray 5 times a day. Medium frequency for Muslims was measured by combining responses for “at least once a week” and “once a month” while low frequency for Muslims captured “at least once a year”, “less than once a year”, and “never”. Christians who attended religious service “everyday” and “at least once a week” were coded as highly frequent because most Christian sects in the region expect attendance only once a week. Medium frequency for Christians was measured by using responses for “at least once a month” while low religiosity for Christians captured “at least once a year”, “less than once a year”, and “never”. The frequency for Muslims and Christians was then combined together to form a variable with three categories; “0” high, “1” medium and “2” low. The belief, frequency and salience variables generated a high reliability coefficient (Cronbach’s alpha = 0.67), which demonstrated high internal consistency of the measure. These variables were combined and a new variable was created from the total of 0–6 scores. High religiosity was measured as categories that ranged from 0 to 4 and low religiosity was categorized as values that ranged from 5 to 6. In other words, highly religious individuals will respond yes to about two of the measures.

Other predictor variables that were considered for this study were age, religious affiliation, gender, educational attainment, parent-child communication, parent’s religious affiliation and presence of a role model. Parent-child communication was measured as “ever discussed sexual matters with mother” and “ever discussed sexual matters with father”. Presence of a role model was included because research has shown that young people’s protective mechanisms may be strengthened by the presence of a role model, which could be a favourite teacher, caring neighbour, religious leader, or even parent or boyfriend or girlfriend at the community level [42]. In this study, role models included were: aunty, brother, family friend, grandparent, other, religious leader, sister, teacher and uncle. Youth who mentioned “other” listed some celebrities as their role models while some of them said their boyfriends were their role models.

### Analytic strategy

Percentage distributions of the demographic and other relevant characteristics of the respondents were carried out using bivariate, as well as multivariate techniques, to identify the relationship between religious affiliation and religiosity on sexual abstinence. In the bivariate analysis, cross-tabulation was made between religious affiliation and both religiosity and sexual abstinence. Pearson’s chi-square test and *p*-values were used to test for the significance. At the multivariate level, we implemented a logistic regression model to examine the associations controlling for other covariates. Analysis was conducted using Stata software (version 15).

## Results

### Descriptive statistics

Participants included 2339 young adults (1311 females, 1028 males) as shown in Table 1. The ages of the respondent ranged from 15 to 24 years with a mean of 20.45 (SD = 2.71). Of the 2339 youth, 29% were catholic and 40% were other Christians. About 30% were Muslim with about 2% practicing traditional religion. More than three quarters (88%) of the youth were highly religious. About 61% of the youth said religion was very important to them compared to 34% who said religion was not important. More than half (62%) of the youth had attained secondary education while about 21% were enrolled in higher education. One third (33%) had worked for pay at some point in their lives. Concerning family characteristics, 82% of the youth were living with their father and less than one fifth (19%) had discussed sexual issues with their fathers. However, about 44% of the youth had discussed sexual issues with their mother. One fifth of the youth had drank alcohol 1 week preceding the interview and about half of the youth reported they had role models. The majority, (68%) of the youth had never had sex compared to 32% of the youth who had done so.

**Table 1:** Frequency Distribution of Characteristics

Demographic Characteristics	Frequency Distribution (%)
Ever had sex	
Yes	758 (32.41)
No	1581 (67.59)
Religiosity	
Low	281 (12.23)
High	2016 (87.77)
Sex	
Female	1311 (56.05)
Male	1028 (43.95)
Age	
15–17	420 (17.96)
18–24	1919 (82.04)
Religious affiliation	
Catholic	671 (28.69)
Other Christian	935 (39.97)
Islam	691 (29.54)
Other	42 (1.80)
Importance of religion	
Important	800 (34.20)
Not important	108 (4.62)
Very important	1431 (61.18)
Parental religion	
Catholic	709 (30.31)
Other Christian	927 (39.63)
Islam	669 (28.60)
Other	34 (1.45)
Education	
No education	72 (3.08)
Primary	322 (13.77)
Secondary	1461 (62.46)
Higher	484 (20.69)
Work for pay	
No	1544 (66.01)
Yes	795 (33.99)
Father alive	
No	510 (21.80)
Yes	1829 (78.20)
Living with father	
No	334 (18.26)
Yes	1495 (81.74)
Ever discuss sex matters with father	
No	1903 (81.36)
Yes	436 (18.64)
Mother alive	
No	307 (13.44)
Yes	1977 (86.56)
Living with mother	
No	326 (16.49)
Yes	1651 (83.51)
Ever discuss sex matters with mother	
No	1309 (55.96)
Yes	1030 (44.04)
Gotten drunk (in last 30 days)	
No	1807 (77.26)
Yes	532 (22.74)
Have role models	
No	1150 (49.17)
Yes	1189 (50.83)
State	
Edo	552 (23.60)
Enugu	638 (27.28)
Osun	623 (26.64)
Kano	526 (22.49)

The percentage distribution of sample characteristics by religiosity is presented in Table 2 to illuminate the characteristics of youth by religiosity. Most of the youth were highly religious in all of the states examined, with Enugu having the highest percentage of high religious youth in comparison to low religious youth (96% vs 4%). In Edo state, 75% of the youth were highly religious compared to 25% of the low religious youth. By education, among the youth with no education, less than half of them were highly religious. However, more than three quarters of youth who were educated were highly religious.

**Table 2:** Distribution of characteristics by religiosity

Demographic Characteristics	Religiosity	
	Low	High
State		
Edo	25.38 (135)	74.62 (397)
Enugu	4.30 (27)	95.70 (601)
Osun	11.92 (74)	88.08 (547)
Kano	8.72 (45)	91.28 (471)
Sex		
Female	13.83 (179)	86.17 (1115)
Male	10.17 (102)	89.83 (901)
Age		
15–17	11.81 (49)	88.19 (366)
18–24	12.33 (232)	87.67 (1650)
Education		
No education	56.34 (40)	43.66 (31)
Primary	19.75 (62)	80.25 (252)
Secondary	8.54 (123)	91.46 (1318)
Higher	11.89 (56)	88.11 (415)
Work for pay		
No	12.99 (198)	87.01 (1326)
Yes	10.74 (83)	89.26 (690)
Father alive		
No	24.19 (120)	75.81 (376)
Yes	8.94 (161)	91.06 (1640)
Living with father		
No	9.88 (32)	90.12 (292)
Yes	8.73 (129)	91.27 (1348)
Mother alive		
No	22.15 (66)	77.85 (232)
Yes	9.54 (186)	90.46 (1764)
Living with mother		
No	10.97 (35)	89.03 (284)
Yes	9.26 (151)	90.74 (1480)
Drink Alcohol		
No	11.44 (203)	88.56 (1571)
Yes	14.91 (78)	85.09 (445)
Have role models		
No	13.21 (149)	86.79 (979)
Yes	11.29 (132)	88.71 (1037)

### Bivariate analysis

Model 1 in Table 3 shows that religiosity is significantly associated with protective youth sexual behaviour. Youth who were highly religious were about two times (OR – 2.04, CI- 1.58-2.63) more likely to abstain from sex.

**Table 3:** Unadjusted and adjusted association between religiosity and sexual abstinence

	Model 1	Model 2	Model 3
Religiosity			
Low			
High	2.04 (1.58–2.63) ***	1.82 (1.24–2.66) ***	1.81 (1.13–2.88) ***
Sex			
Female			
Male	0.84 (0.71–1.00)		1.09 (0.83–1.45)
Age			
15–17			
18–24	0.15 (0.10–0.22) ***		0.19 (0.11–0.32) ***
Religious affiliation			
Catholic			
Other Christian	0.72 (0.58–0.89) ***		0.81 (0.49–1.35)
Islam	1.38 (1.09–1.75) ***		0.39 (0.14–1.05)
Other	0.66 (0.35–1.26)		
Parental religion			
Catholic			
Other Christian	0.77 (0.63–0.95) *		0.89 (0.54–1.48)
Islam	1.78 (1.40–2.27) ***		3.61 (1.41–9.22) ***
Other	0.71 (0.35–1.43)		2.66 (0.19–35.90)
Education			
No education			
Primary	6.08(3.51–10.53) ***		2.25 (0.61–8.28)
Secondary	4.88 (2.96–8.04) ***		2.97 (0.88–10.00)
Higher	2.09 (1.24–3.51) ***		1.94 (0.57–6.60)
Work for pay			
No			
Yes	0.47 (0.35–0.63) ***		0.74 (0.55–0.99)
Living with father			
No			
Yes	2.99 (2.35–3.82) ***	2.62 (1.90–3.60) ***	1.93 (1.32–2.82)
Ever discuss sex matters with father			
No			
Yes	0.72 (0.58–0.90) ***		0.91 (0.65–1.28)
Living with mother			
No			
Yes	2.83 (2.22–3.61) ***	1.72 (1.21–2.45) ***	2.12 (1.39–3.23) ***
Ever discuss sex matters with mother			
No			
Yes	0.70 (0.59–0.83) ***		0.63 (0.47–0.84) ***
Drink Alcohol			
No			
Yes	0.16 (0.13–0.20) ***		0.19 (0.13–0.26) ***
Have role models			
No			
Yes	1.13 (0.95–1.35)	1.41 (1.13–1.77) ***	1.66 (1.28–2.16) ***
State			
Edo			
Enugu	1.52 (1.20–1.94) ***		2.36 (1.57–3.54) ***
Osun	1.46 (1.15–1.86) ***		1.45 (0.85–2.56)
Kano	1.76 (1.36–2.28) ***		2.06 (1.37–3.10) ***

*p < 0.1; **p < 0.05; ***p < 0.01. Blank items are reference categories

Other covariates were also associated with youth sexual behaviour at the bivariate level. For instance, older youth aged 18–24 (OR – 0.15, CI- 0.10-0.22) had significantly lower odds of abstaining compared to their counterparts 15–17. Youth who were other Christians (OR – 0.72, CI- 0.58-0.89) had significantly lower odds of abstaining compared to Catholic youth; however, Muslim youth (OR – 1.38, CI- 1.09-1.75) were significantly more likely to abstain compared to Catholic. The direction of associations were similar for parental religion and youth sexual abstinence.

By socio-economic characteristics, youth that were enrolled in higher education (OR – 2.09, CI- 1.24-2.27) had significantly higher odds of abstaining while youth who were working for pay were less likely to abstain (OR – 0.47, CI- 0.35-0.63).

Living arrangement, parent child communication, and alcohol use were other variables significantly associated with youth sexual behaviour at the bivariate level. For instance, youth living with father and youth living with mother had significantly higher odds of sexual abstinence. However, discussing sex related matters with father and mother was associated with a lower likelihood of sexual abstinence among youth.

### Multivariate analysis

In Table 3, we present data that examines whether parental religion and presence of parents in the household changes the effect of religiosity on sexual abstinence. The association between religiosity and youth sexual behaviour remain unchanged. In the final model, which included religiosity and all the covariates, religiosity remained a significant factor for youth abstinence.

Youth who were highly religious had higher odds (OR – 1.81, CI- 1.13-2.88) of abstaining from sex compared to their counterparts with low levels of religiosity. Older youth aged 18–24 (OR – 0.19, CI- 0.11-0.32) had significantly lower odds of abstaining. Youth who reported that their parents practiced Islam (OR – 3.61, CI- 1.41-9.22) were significantly more likely to be abstaining.

Youth who were living with mother alone had high odds of abstaining (OR – 2.12, CI- 1.39-3.23) but youth who discussed sexual issues with their mother (OR – 0.63, CI- 0.47-0.84) were significantly less likely to abstain. These associations were reversed for youth living with father alone. Alcohol use (OR – 0.19, CI- 0.13-0.26) showed a negative association with abstinence. Having role models became significant in the full model. Youth who had role models (OR – 1.66, CI- 1.28-2.16) were about two times more likely to abstain from sex.

There was no significant association between youth sexual behaviour and gender. There was no significant association between sexual behaviour and education, work status, living with father or ever having discussed sexual issues with father. The association between religious affiliation and youth sexual behaviour in the unadjusted model was not seen in the adjusted model.

The results in Table 4 present the association between religiosity and parental religion and this association was not significant.

**Table 4:** Interaction terms between Religiosity and Parental Religion

Main effects (Religiosity)	Other Christian	Islam	Others
High	0.82	0.91	0.47

## Discussion

Religiosity has been documented to be a protective factor in the lives of young people, so this study examined the association between religiosity and youth sexual behaviour as measured by abstinence. The study also examined whether this association changed in the presence of parental religion or presence of a parent in the household. Results indicate that religion is indeed an important aspect for the sexual behaviour of young people in Nigeria. Interventions aimed at impacting youth sexual behaviour might therefore benefit from a strength-based approach that underscores the importance of religion and parental resources present in the lives of youth.

A large number of the youth (68%) in this study report that they have never had sex. This result shows that young people are engaging in positive sexual behaviour and this pattern should not be ignored. Our results are similar to other studies in sub-Saharan Africa [43] and in Nigeria [44]. These findings are also similar to the recent Nigerian Demographic Health Survey. Young people are abstaining from sex, which is very important for their health and sexual development. Although there are debates surrounding abstinence only messages [45], this behaviour still remains one of the most effective methods of avoiding unplanned pregnancy. Sustaining strategies that have led to young people delaying sexual debut and abstaining is crucial for the sexual and reproductive health of youth.

There was a positive relationship between religiosity and youth sexual abstinence in the unadjusted model. Youth who were highly religious had higher odds of abstaining and this association remained the same in the adjusted model. Our findings are in support of the theoretical framework and hypothesis. They are also in support of other studies in developed [30] and developing countries [12].

The religiosity of the youth may influence their decision to abstain directly based on messages they listen to at their place of worship. Youth who are more religious have a higher likelihood of adjusting to the values and norms of their place of worship. Another way in which religiosity could influence the behaviours of youth could be based on fear of sanctions placed by religious groups or leaders. Nigeria is very religious and various denominations enforce particular norms such as mode of dress, which may help protect youth from behaviours that place them at risk. Some youth who have unwanted pregnancy may be ostracized from particular groups in the church [46]. There was no difference in sexual behaviour of Muslim and Christian youth. This could be as a result of their similar stance on premarital sex among young people.

Indirectly, religiosity of youth could influence their sexual behaviour because of the time spent at the various place of worship and the activities they may engage in at these places. For instance, some churches may need the skills of young people for capacity development. These activities could be in form of workshops that are being organized for youth by the religious group. In addition, some religious organizations serve as sources of social capital for young people by sponsoring their education or helping them acquire some set skills that may keep them from risky behaviours.

At the individual level, gender was not associated with sexual abstinence among the youth independently. The effects of religiosity on abstinence did not differ by sex. Our results do not support our first hypothesis that the effect of religiosity on sexual abstinence would differ by gender. This is unexpected as religiosity could reinforce traditional gender norms, which expects women to abstain and men to be promiscuous.

Our results confirm the other hypotheses. The presence of parents in the household was also associated with abstinence among youth. Having a parent present may allow for parental monitoring, which may reduce the chances of youth engaging in risky behaviours. The positive association between role models and youth sexual behaviour cannot be overlooked. These role models could also be religious leaders and youth may be inclined to follow the behaviours of their models.

The strengths of this study include the relevant research question, the adequate sample size for testing the hypotheses and the sampling procedure.

### Implications for policy

Religiously was strongly associated with youth sexual behaviour among youth sampled in this study. This suggests that policy makers and community advocates could present youth with various religious events that can improve their spiritual development. These activities could be in form of active youth groups that engage in sports, music or arts. Older youth in these religious spaces should also be readily available to mentor younger youth and support them in other ways which could be in form of financial support. This may protect their exposure to risky behaviours. The results on gender are important for policy as it is possible that religious beliefs could be used to change traditional gender norms that encourage male promiscuity.

## Conclusion

This study adds to the literature by exploring the association between youth religiosity and their protective sexual behaviour. We also examined whether this association remains significant in the presence of parents, parental religion and other characteristics. This is important for policy makers, as religion can serve as a tool for behavioural change. Family demographers can study the continuity of parental religion where religious patterns are changing rapidly. Therefore, program planners may want to design and implement preventive interventions aimed at improving or sustaining healthy youth behaviours.

### Limitations

Although we tried to sample four states from the different regions in Nigeria, which may be broadly representative of Nigeria, they are only four of the 36 states in the country. The sexual behaviours of the youth in other states may differ. Also, the study was conducted among youth recruited using non-probability sampling methods from different locations where youth congregate and we could not obtain cluster sizes for youth in these locations. The outcome variable may have been influenced by social desirability bias as women are expected to remain virgins before marriage in most parts of the country. In addition, the study design is cross-sectional and was not able to establish how other events may have influenced youth sexual behaviour during their life course. Nevertheless, the use of ODK helped put the participants at ease as they filled the questionnaires on the phones themselves which ensured confidentiality and not having to verbalize their responses.

### Additional file

Additional file 1: Protective Sexual Behaviour. (XLSX 52 kb)

## Appendix

**Table 5:** Sample size by state

State	Sample Size
Edo	552
Enugu	638
Osun	623
Kano	526

## Abbreviations

DHS: Demographic health survey
ODK: Open data kit
